# Porcine blood cell and brain tissue energy metabolism: Effects of “early life stress”

**DOI:** 10.3389/fmolb.2023.1113570

**Published:** 2023-04-17

**Authors:** Franziska Münz, Eva-Maria Wolfschmitt, Fabian Zink, Nadja Abele, Melanie Hogg, Andrea Hoffmann, Michael Gröger, Enrico Calzia, Christiane Waller, Peter Radermacher, Tamara Merz

**Affiliations:** ^1^ Institute for Anesthesiological Pathophysiology and Process Engineering, Ulm University Medical Center, Ulm, Germany; ^2^ Clinic for Anesthesiology and Intensive Care, Ulm University Medical Center, Ulm, Germany; ^3^ Department of Psychosomatic Medicine and Psychotherapy, Nuremberg General Hospital, Paracelsus Medical University, Nuremberg, Germany

**Keywords:** high resolution respirometry, mitochondrial respiration, electron spin resonance, superoxide anion, PBMC, granulocyte

## Abstract

**Background:** Early Life Stress (ELS) may exert long-lasting biological effects, e.g., on PBMC energy metabolism and mitochondrial respiration. Data on its effect on brain tissue mitochondrial respiration is scarce, and it is unclear whether blood cell mitochondrial activity mirrors that of brain tissue. This study investigated blood immune cell and brain tissue mitochondrial respiratory activity in a porcine ELS model.

**Methods:** This prospective randomized, controlled, animal investigation comprised 12 German Large White swine of either sex, which were weaned at PND (postnatal day) 28–35 (control) or PND21 (ELS). At 20–24 weeks, animals were anesthetized, mechanically ventilated and surgically instrumented. We determined serum hormone, cytokine, and “brain injury marker” levels, superoxide anion (O_2_^•^¯) formation and mitochondrial respiration in isolated immune cells and immediate *post mortem* frontal cortex brain tissue.

**Results:** ELS animals presented with higher glucose levels, lower mean arterial pressure. Most determined serum factors did not differ. In male controls, TNFα and IL-10 levels were both higher than in female controls as well as, no matter the gender in ELS animals. MAP-2, GFAP, and NSE were also higher in male controls than in the other three groups. Neither PBMC routine respiration and brain tissue oxidative phosphorylation nor maximal electron transfer capacity in the uncoupled state (ETC) showed any difference between ELS and controls. There was no significant relation between brain tissue and PBMC, ETC, or brain tissue, ETC, and PBMC bioenergetic health index. Whole blood O_2_^•^¯ concentrations and PBMC O_2_^•^¯ production were comparable between groups. However, granulocyte O_2_^•^¯ production after stimulation with *E. coli* was lower in the ELS group, and this effect was sex-specific: increased O_2_^•^¯ production increased upon stimulation in all control animals, which was abolished in the female ELS swine.

**Conclusion:** This study provides evidence that ELS *i*) may, gender-specifically, affect the immune response to general anesthesia as well as O_2_^•^¯ radical production at sexual maturity, *ii*) has limited effects on brain and peripheral blood immune cell mitochondrial respiratory activity, and *iii*) mitochondrial respiratory activity of peripheral blood immune cells and brain tissue do not correlate.

## 1 Introduction

Childhood maltreatment (CM) (neglect, physical, emotional or sexual abuse) significantly increases the risk to develop chronic somatic and/mental diseases (Felitti et al., 1998; Nemeroff 2016; World Health Organization, 2022). In addition, CM may exert various direct biological effects: for example, patients with a history of Early Life Stress (ELS) present with activation of peripheral blood mononuclear cells (PBMC), in particular T cells (Elwenspoek et al., 2017). In experimental animals, this ELS-related systemic pro-inflammatory status coincides with chronic neuro-inflammation (González-Pardo et al., 2020; Roqué et al., 2016). Moreover, ELS is associated with increased oxidative stress, both in preclinical models (Ho et al., 2016; Réus et al., 2017) as well as in patients (Boeck et al., 2016; Boeck et al., 2019; Horn et al., 2019). However, equivocal data is available on the impact of this aggravated level of oxidative stress on mitochondrial respiration: in animals, ELS-induced oxidative stress was associated with mitochondrial dysfunction (Amini-Khoei et al., 2017; González-Pardo et al., 2020), while patients with CM showed even higher mitochondrial activity in PBMC when compared to a control group (Boeck et al., 2016; Boeck et al., 2018; Gumpp et al., 2020; Gumpp et al., 2022). On the one hand, these conflicting results may be due to the fact that upon activation, peripheral immune cells show metabolic plasticity with modifications of mitochondrial respiratory activity, which, moreover, is cell type-specific with respect to its extent and duration (Pearce and Pearce 2013; Pearce et al., 2013; Zhang et al., 2020). Furthermore, it remains an open question whether immune cell energy metabolism of peripheral, blood borne, immune cells represents that of brain tissue cells, in particular microglia (Culmsee et al., 2019), which are hardly accessible. Clearly, as mentioned-above, statistically significant associations between the presence and/or severity of ELS and alterations of PBMC mitochondrial respiratory activity have been demonstrated (Boeck et al., 2016; Boeck et al., 2018; Gumpp et al., 2020; Gumpp et al., 2022), but only scarce data are available on the relation between blood-based and brain energy metabolism: so far, to the best of our knowledge, a study of non-human primates is the only one to directly compare mitochondrial respiration of monocytes and isolated frontal cortex mitochondria. The authors concluded that their data provide evidence that “*blood-based bioenergetics profiling can serve as a minimally invasive measure of systemic bioenergetic capacity that is positively related to measures of brain mitochondrial function and metabolism*”, but “*is not a surrogate for direct measures of brain metabolism*” (Tyrrell et al., 2017).

Based on these considerations, the aim of this study was to investigate whether *i*) brain and peripheral blood immune cell bioenergetic profiles are related to each other, and *ii*) ELS may affect these profiles in 6-month-old pigs.

## 2 Materials and methods

### 2.1 Animals

The experiments were performed after obtaining the approval by the University of Ulm Animal Care Committee and the Federal Authorities for Animal Research (Regierungspräsidium Tübingen; Reg.-Nr. 1559, approval 29 October 2021) and in compliance with the National Institute of Health Guidelines on the Use of Laboratory Animals and the European Union “Directive 2010/63/EU on the protection of animals used for scientific purposes”. The data presented are from twelve young sexually mature (median [interquartile range] age 23 [22; 24] weeks, bodyweight 82 [67; 93] kg) German Large White pigs with equal sex distribution (n = 3 males/females each per group). Animals of the “control” group had been weaned at day 28–35 after birth, which corresponds to the weaning period regularly used for swine husbandry. In contrast, swine with “Early Life Stress (ELS)” had already been weaned at day 21 after birth. This time point was chosen, because *i*) it represents the earliest time point for swine weaning described in the Federal German regulations on farm animal husbandry (“Tierschutz-Nutztierhaltungsverordnung–TierSchNutztV, 22 August 2006; last amendment 29 January 2021), and *ii*) has been evaluated as “…*not to cause any violation of animal protection …* " according to the chapter no. 90 entitled *“Influence of weaning age on piglet behavior*” (“*Einfluss des Absetzalters auf das Verhalten von Ferkeln nach dem Absetzen*”) of the report on “*Environmentally compatible and site-specific agriculture*” (“*Umweltverträgliche und Standortgerechte Landwirtschaft*”) of the Friedrich-Wilhelms-University, Bonn, Germany. Moreover, *iii*) according to the literature (Smith et al., 2010) the 21 days weaning timepoint induces a mild stress phenotype in contrast to weaning at 28–35 days and *iv*) we aimed at avoiding any pathological clinical symptoms associated with earlier (at day 16–18) weaning of piglets in other ELS models, e.g., diarrhea, weight loss, and/or intestinal mucosal barrier dysfunction (Pohl et al., 2017; McLamb et al., 2013; Moeser et al., 2007; Smith et al., 2010). The acute response to early weaning has previously been investigated by Moeser et al., 2007: They focused on the effects of early weaning on intestinal function and determined intestinal barrier dysfunction and CRF receptor dysregulation induced by weaning. Smith *et al.* performed a time course experiment focusing on the effects of different early weaning ages (15, 18, 21, 23 and 28 days of age) on intestinal function and determined that the earlier the weaning the more detrimental to intestinal function at 35 days of age; moreover, there were clear differences in intestinal function between piglets weaned at 21 vs 28 days of age (Smith et al., 2010). Differences between early (15 days) and later weaned (23 days) pigs were still detectable at 9 weeks of age (Smith et al., 2010). We chose a rather mild phenotype of ELS with only 1 week of difference between early weaning and the control group, because even earlier weaning is associated with the development of an “irritable bowel syndrome”-like phenotype in older pigs (Pohl et al., 2017), thus these animals suffer from frequent diarrhea, which significantly changes their overall physiology and fluid balance. We wanted to avoid such “obvious” disturbance factors as covert hypovolemia, changes in electrolytes and acid-base-balance, which might also be associated with hemodynamic instability and arrhythmias in response to anesthesia, in particular during the induction phase. Thus, we decided to use 21 days as our early weaning timepoint, which reportedly leads to mild phenotypical changes, but is not associated with major physiological disturbances.

The animals were obtained from an agricultural production specified in breeding and rearing of piglets (Schweinezucht Kugler Gmbh & Co. Kg, Ostrach, Germany). This is also where the weaning was performed. The breeding facility is located approx. 100 km from our research facility, which helped to keep the transport of the animals as short as possible. The animals were kept at the breeding facility for up to 2–4 weeks before the experiments. After weaning, the piglets were kept in groups of 42 mixed-sex groups on polymer concrete, with access to a fodder rack with straw and were fed with fodder produced by the breeder. When the piglets reached approx. 30 kg of weight they were transferred to the subadult area where they were kept in groups of a maximum number of 190 animals divided in sections of a maximum number of 14 animals per group and separated by sex. Animals from different piglet groups were not mixed to prevent them from having to fight for a new hierarchy. The sub-adult area had a concrete slatted floor with access to a fodder rack with hay/straw/silage and animals were fed with fodder produced by the breeder. After transfer to our facility, the animals were kept separated by sex in separate boxes, but in contact to the neighboring boxes. They had regular run and were provided activity by the animal caretaker. They received the same fodder as before, provided by the breeder, additionally they had *ad libitum* access to hay and straw. Male and female animals were kept under the same conditions. Against the common practice of the breeder, male animals were specifically not castrated to allow for higher translational relevance and more distinct evaluation of gender-dependent effects.

At the breeding facility, regular health monitoring by the “Schweinegesundheitsdienst SGD” (pig health service) and the “Tierseuchenkasse” (animal plague office) was performed. After transfer to our facility, an incoming control comprised checking for normal breeding and behavior typical for pigs. In case of irregularities, the single animal was evaluated, and stool samples from all animals were checked for parasites.

Neither the most dominant animals nor the runts of the litter were included in the experiments. Fights for the hierarchy were prevented by not mixing animals from different established social groups. Furthermore, the animals were kept in age-specific groups, and animals from different ages were not mixed. In order to minimize inter-individual differences with respect to age and development, pairs of one control and one ELS animal, respectively, were taken from the same litter.

### 2.2 Anesthesia and surgery

After their last meal at the evening before the experiment, the animals had free access to water and were kept without bedding in the last 12 h before the experiment. In the morning of the experimental day, the pigs received an intramuscular pre-medication comprising azaperone (2 mg/kg) and midazolam (0.5–1 mg/kg); being deeply asleep, an intravenous access was established *via* an ear vein. Thereafter, general total i. v. Anesthesia was induced using propofol (1.5–2.0 mg/kg) and ketamine (1 mg/kg), followed by endotracheal intubation. Then, i. v. Fentanyl was administered (20 μg/kg), and muscle paralysis was achieved using pancuronium (0.1 mg/kg). The animals were mechanically ventilated using the following ventilator settings: tidal volume 8 mL/kg, respiratory rate 8–12 breaths/minute adapted to achieve an arterial PCO_2_ (PaCO_2_) = 35–40 mmHg, inspiratory/expiratory ratio (I/E) of 1:1.5, fraction of inspiratory oxygen (F_i_O_2_) of 0.3, positive end-expiratory pressure 10 cmH_2_O to prevent atelectasis formation (Datzmann et al., 2022; Datzmann et al., 2021). Anesthesia was maintained by continuous i. v. Infusion of propofol (10 mg/kg/h). A balanced electrolyte solution (10 mL/kg/h, Jonosteril^®^ 1/1^®^, Fresenius Kabi) was infused as maintenance fluid. A 10F-metal-sheathed catheter (Arrow^®^) for continuous blood pressure monitoring and blood sampling was placed in the left iliac artery *via* a surgical cut down. In addition, in order to maintain adequate systemic hemodynamics, atropine was administered to counteract bradycardia <55 beats/min, Akrinor^®^ (i.e., combined theodrenaline/cafedrine 10/200 mg/mL, ratiopharm, Ulm) to maintain mean arterial pressure (MAP) > 70 mmHg. Immediately after completion of the surgical instrumentation, ventilator settings were modified to an I/E ratio = 1:2, F_i_O_2_ = 0.21, and zero end-expiratory pressure (0 cmH_2_O) to closely mimic physiological conditions.

### 2.3 Experimental protocol

In order to avoid any effect of circadian rhythm, all experiments followed a strict timeline. Blood sampling under anesthesia is inevitable in a pig experiment, especially when trying to determine stress-related effects. Taking a blood sample in an awake pig would induce significant acute psychological stress potentially overriding the subtler effects induced by the early weaning. The surgical instrumentation was necessary, because rather large quantities of blood are needed to be able to purify enough immune cells to be able to determine mitochondrial respiratory capacity, which cannot be taken by a simpler method. The surgical instrumentation also allowed us to provide treatment for hemodynamic stabilization to the pigs during blood sampling (fluid administration, Akrinor^®^, *etc.*). The actually measured catecholamine plasma concentrations in the animals were in the lower physiological range, suggesting that anesthesia prevented any major surgery-induced significant stress response. Intramuscular pre-medication was performed at 6:00 a.m., induction of general anesthesia at 7:00 a.m., followed by approx. 35–45 min of surgical instrumentation. A first blood sampling was performed after a 30-min recovery period, the second (terminal) blood sampling took place another 30 min later after compensation of the blood sampling-related blood loss with synthetic gelatin (Gelafundin^®^, Braun, Melsungen). Thus, the anesthesia and surgery were performed consistently and within the same timeline in all animals. As a consequence, even though a “naïve” baseline was not determined, a comparison of the results between the experimental groups is still useful and valid. Finally, Thereafter, the pigs were euthanized with KCl after further deepening of the anesthesia (0.5 mg fentanyl i. v.) for immediate *post mortem* brain preservation. In between the individual experiments, the timeline varied <15 min.

Immediately *post mortem*, the head of the pig was removed. A skin incision on the middle of the forehead of the pig was performed, and the skin and muscle tissue were removed from the skull. The top of the skull was removed with the help of a bone saw and chisel. The dura mater was carefully opened with a scalpel. Afterwards, the pig head was balanced on the snout to facilitate the severing of the cranial nerves, after which the brain fell out of the skull and was gently caught by hand. Immediately thereafter, fresh tissue samples from the prefrontal cortex were transferred to Custodiol^®^ (Dr. Franz Köhler Chemie GmbH, Bensheim, Germany) for the analysis of mitochondrial respiratory capacity. The removal of the brain was always performed by the same trained and experienced veterinarian (AH). The time between the death of the animal and brain sampling was consistent for all experiments (30 ± 5 min).

### 2.3 Measurements and calculations

At the two measurement points, in addition to recording of blood temperature, heart rate and MAP, arterial blood samples were taken for the measurement of PaO_2_ and PaCO_2_, acid-base-status, electrolyte (Na^+^, K^+^) levels and metabolic parameters (lactate, glucose). These parameters were measured using the automatic blood gas analyzer which not only provides blood gas tensions but also acid-base status, electrolyte concentrations as well as lactatemia and glycemia. Furthermore, at the first blood sampling after the 30-min recovery period, a blood cell count was obtained with the help of a Neubauer chamber and an Automated Cell Counter (BioRad TC20) corrected for swine species-specific cell size. At the same time-point, cytokines (tumor necrosis factor, interleukin-6 and 10), renin, aldosterone, troponin, testosterone, ACTH and “brain injury markers” were measured using pig-specific commercially available kits (cytokines: Quantikine ELISA Porcine, R&D Systems; Troponin: Pig Cardiac Troponin I, Life Diagnostics; Testosterone: Pig Testosterone, Abnova; ACTH: Pig ACTH ELISA kit, Abbexa; brain markers, Renin, Aldosterone: Porcine ELISA kits, BlueGene BioTech). 8-Isoprotane was determined with an EIA kit (Cayman Chemical). Cortisol values were determined with a commercially available kit for human plasma (IBL International), since it does not differ between species. Catecholamine levels (adrenaline, noradrenaline) were determined after centrifugation of whole blood samples in Li^+^-heparine-coated, stabilizor-primed (20 μL/mL blood containing 0.2 M reduced glutathione and 0.2 M ethylenglycol-bis(aminoethylether)-N,N,N′,N′-tetra-acetic acid (EGTA), both Carl-Roth, Karlsruhe) tubes using liquid-chromatography/tandem-mass-spectrometry (LC-MS/MS) by a service lab (Dr. Eberhard & Partner, Dortmund).

Isolation of peripheral blood mononuclear cells (PBMC) and granulocytes was performed as described recently (Wolfschmitt et al., 2023). For this purpose, approx. 300 mL of arterial whole blood were collected in 10 mL-LiHep monovettes (Sarstedt, Nümbrecht) from the catheter placed in the left iliac artery. After sampling, the blood was diluted 1:1 with phosphate-buffered saline (PBS; without CaCl_2_, MgCl_2_) and carefully layered onto two density gradient solutions (9 mL 1.119 g/L and 8 mL 1.088 g/L solution, Pancoll, PAN Biotech, Aidenbach) before centrifugation at 764 *g* for 20 min without break at room temperature (RT). Density centrifugation yielded a buffy coat containing PBMCs and a bottom layer comprising red blood cells (RBCs) and granulocytes. After washing with 1× PBS, the buffy coat was subjected to osmotic lysis to remove residual RBCs. The lysis was stopped with 10× PBS, and PBMCs were washed and resuspended in 1× PBS before counting cells in a Neubauer counting chamber. In order to purify granulocytes from the bottom layer of the density gradient solution, osmotic lysis had to be performed a total of three times to eliminate RBC contamination. Analogously to PBMCs, granulocytes were washed with 1× PBS after lysis and subsequently counted.

Mitochondrial respiration in the isolated immune cells as well as the brain tissue specimens (pre-frontal cortex) was measured by high-resolution respirometry using the Oxygraph-2K^®^ (Oroboros Instruments, Innsbruck) as described previously (Datzmann et al., 2023; Datzmann et al., 2021; Zink et al., 2021; Wolfschmitt et al., 2023) This device allows for simultaneous recording of the O_2_ concentration in two parallel chambers calibrated for 2 mL of respiration medium MiR05 (Doerrier et al., 2018; Zink et al., 2021). This medium is composed of 110 mM D-Sucrose (Sigma Aldrich, St. Louis, MO, United States), 60 mM K-Lactobionate (Sigma Aldrich, St. Louis, MO), 0.5 mM ethylene glycol tetra acetic acid (Sigma Aldrich, St. Louis, MO), 1 g/L bovine serum albumin free from essentially fatty acids (Sigma Aldrich, St. Louis, MO), 3 mM MgCl_2_ (Scharlau, Hamburg), 20 mM taurine (Sigma Aldrich, St. Louis, MO), 10 mM KH_2_PO_4_ (Merck, Darmstadt), 20 mM HEPES (Sigma Aldrich, St. Louis, MO), adjusted to pH = 7.1 with KOH and equilibrated with 21% O_2_ at 37°C. Cell suspensions or brain tissue homogenates containing 10 × 10^6^ cells/mL or 1 mg tissue/mL of respiration medium were filled into both chambers and continuously stirred at 750 rpm. Closing the chambers by gently pushing down the stoppers started the continuous recording of mitochondrial respiration, which was quantified in terms of O_2_ flux (J*O*_*2*_) based on the rate of change of the O_2_-concentration in the chambers normalized for cell number or tissue weight. Once the chambers were sealed, specific analysis of mitochondrial respiratory function was achieved by sequential injections of substrates and inhibitors into the respiration medium. For blood cell samples, firstly, routine respiration was recorded once a stable J*O*_*2*_-value was achieved after closing the chambers. Afterwards, 2.5 μM oligomycin was injected to block the ATP-synthase. This yielded the LEAK-state, which represents the respiratory activity required to maintain a stable membrane potential in absence of ATP-turnover. Thereafter, the titration of carbonyl cyanide p-(trifluoromethoxy)-phenylhydrazone (FCCP) in 1 µM steps allowed to achieve the respiratory activity in the un-coupled state. The maximum respiratory capacity (ETC.) was determined after the addition of 2 mM malate, 10 mM glutamate, 5 mM ADP, 5 µM cytochrome c, 10 mM pyruvate and 10 mM succinate. Finally, 0.5 μM rotenone +5 μM antimycin were added to block complex I and III respectively, yielding the residual (non-mitochondrial) O_2_ consumption. For tissue samples, the maximum respiratory capacity in the coupled state (OxPhos) was determined after the addition of 2 mM malate, 10 mM glutamate, 5 mM ADP, 5 µM cytochrome c, 10 mM pyruvate, 1 mM octanoyl-carnitine and 10 mM succinate. The LEAK state was recorded after the addition of 2.5 µM oligomycine, and the maximum respiratory capacity in the uncoupled state (ETC.) was measured after the titration of 1 µM FCCP. The data shown are normalized for tissue wet weight and cell number for the brain specimens and isolated blood immune cell count, respectively. Finally, the “Bioenergetic Health Index (BHI)" was calculated as described by Chacko et al. (2014) according to the formula:

$$BHI=\log\frac{\left(reserve\;capacity\times ATP-linked\;O_{2}\;consumption\right)}{\left(non-mitochondrial\;O_{2}consumption\times LEAK\;respiration\right)}$$

Whole blood superoxide anion (O_2_^•−^) concentrations were determined immediately after sampling as described previously (Zink et al., 2021; Datzmann et al., 2022; Datzmann et al., 2023). For this purpose, 25 µL of whole blood were mixed with an aliquot of 25 µL freshly thawed CMH spin probe solution. The CMH solution contained 400 µM CMH spin probe (1-Hydroxy-3-methoxycarbonyl-2,2,5,5-tetramethylpyrrolidine), 25 µM deferoxamine, and 5 µM diethyldithiocarbamate to chelate transition metal ions in Krebs-HEPES-Buffer (KHB) (Noxygen, Elzach, Germany). After mixing whole blood with CMH, the solution was transferred to a 50 µL glass capillary, sealed, and measured with an EMXnano electron spin resonance (ESR) spectrometer (Bruker, Billerica, MA, United States) after 5 min incubation at 37°C (Bio-III, Noxygen, Elzach, Germany). For each measurement, 3 scans of the following settings were averaged: 3440 G center field, 60 G sweep width, 72.70 ms conversion time, 9.66 GHz microwave frequency, 0.3162 mW microwave power, and 2 G modulation amplitude. Radical concentration was quantified by comparison with a series of CP^•^ radical standards solved in KHB. As a blank sample, KHB added to the respective amount of CMH was measured and subtracted from the sample value. For the determination of immune cell O_2_^•−^ production (Wolfschmitt et al., 2023), 25 µL of a cell suspension containing 2.5 × 106 cells/mL purified granulocytes and mononuclear cells were transferred to FACS tubes and washed in RPMI 1640 medium (Glucose 1.8 mg/mL, Glutamine 0.6 mg/mL, NaHCO_3_ 100 μg/mL, HEPES 20 mM) while being kept on ice. 50 μL of 1 mg/mL pHrodo^®^ *E. coli* BioParticles^®^ Conjugate (Life technologies TM Carlsbad, CA) was added to the cell suspension before subsequent incubation at 37°C for exactly 20 min. Reactions were stopped by transferring cells on ice and adding 1 mL of washing buffer (pHrodoTM Bioparticles Phagocytosis Kit for Flow Cytometry, Life technologies TM Carlsbad, CA). PBMCs and granulocytes were washed once with PBS before being transferred to a 1.5 mL tube and being resuspended in 1 mL RPMI. For the subsequent ESR analysis, the cell suspension was mixed with 25 µL of CMH. In contrast to whole blood, cell samples were measured 8 times over 30 min to calculate the O_2_^•−^ production rate. Otherwise, the ESR settings were the same as used for a single whole blood measurement. A sample of RPMI 1640 medium mixed 1:1 with CMH was used as a blank value for measuring cell suspensions and subtracted from sample values. Data were evaluated with the Xenon_nano software (Bruker BioSpin, Rheinstetten).

### 2.5 Data analysis

All data are presented as median (interquartile range) unless stated differently. We had predefined the activity of the brain tissue mitochondrial respiratory chain as the main outcome parameter; the methodology had already been used and validated in porcine brain tissue samples previously (Datzmann et al., 2022; Datzmann et al., 2021). However, in those studies, none of the interventions studied had significantly affected mitochondrial respiration. Consequently, a power analysis on the possible effect of ELS had not been feasible, due to *i*) the unavailability of appropriate data, and the fact that, as mentioned above, *ii*) the time point of early weaning had been chosen to avoid symptoms associated with earlier (at day 16–18) weaning of piglets in other ELS models, e.g., diarrhea, weight loss, and/or intestinal mucosal barrier dysfunction (Moeser et al., 2007; Smith et al., 2010; McLamb et al., 2013; Pohl et al., 2017). Hence, we were unable to calculate a number of animals to include to observe a difference in mitochondrial respiratory activity. Therefore, the Animal Care Committee of the Universität Ulm and the Federal Authorities for Animal Research (Regierungspräsidium Tübingen) deemed our study as “exploratory” allowing for n = 6 per group only. For the complete set of data, normal distribution was tested by Shapiro-Wilk test. Thereafter, intergroup differences were tested using an unpaired Student’s t-test or a Mann-Whitney *U*-test as appropriate. Statistical evaluations focusing on sex-effects were analyzed with two-way ANOVA. Correlation coefficients were calculated according to Pearson. Statistical analysis was carried out with GraphPad Prism5^®^ (GraphPad Software Inc., San Diego, CA).

## 3 Results

Tables 1–3 summarize the clinical observations as well as the parameters measured of hemodynamics, gas exchange, acid-base status, metabolism (Table 1), administered drugs (Table 2) and blood cell count (Table 3). At the end of the experiment, ELS animals presented with a tendency towards lower MAP (see Table 1) despite higher need for circulatory support (see Table 2) and higher glycemia levels than the control group (see Table 1). In addition, 3/6 ELS animals vs 1/6 control pigs showed cardiac arrhythmia (i.e., ventricular extrasystole and/or atrio-ventricular block II°). None of the other parameters showed any intergroup difference.

**TABLE 1:** Parameters of systemic hemodynamics, gas exchange, acid-base-status, and metabolism in the control and Early Life Stress (ELS) groups (n = 6 each). F_i_O_2_: inspired O_2_ fraction; K^+^: potassium; MAP: mean arterial pressure; Na^+^: sodium; PaCO_2_: arterial CO_2_ partial pressure; PaO_2_: arterial O_2_ partial pressure; all data are median (interquartile range); bold numbers refer to *p*-values for intergroup comparison.

		After instrumentation and 30 min recovery period	Terminal blood sampling
Body temperature [°C]	Control	36.4 (36.2; 37.1)	36.2 (35.9; 36.8)
	ELS	36.7 (36.5; 37.0)	36.6 (36.5; 36.8)
Heart rate [1/min]	Control	69 (61; 70)	64 (59; 74)
	ELS	71 (63; 75)	73 (66; 78)
MAP [mmHg]	Control	86 (85; 89)	85 (76; 93)
	ELS	75 (73; 81)	74 (69; 83) **p = 0.057**
PO_2_ [mmHg]	Control	94 (87; 125)	84 (81; 124)
	ELS	94 (88; 99)	97 (84; 100)
PaO_2_/F_i_O_2_	Control	425 (390; 449)	387 (381; 408)
	ELS	423 (415; 431)	406 (382; 449)
PaCO_2_ [mmHg]	Control	37 (34; 39)	37 (37; 38)
	ELS	38 (37; 38)	37 (34; 39)
pH	Control	7.44 (7.44; 7.46)	7.45 (7.43; 7.46)
	ELS	7.44 (7.43; 7.48)	7.45 (7.43; 7.46)
Arterial base excess [mmol/L]	Control	1.8 (0.3; 2.0)	1.4 (1.2; 1.8)
	ELS	1.8 (1.0; 4.0)	1.6 (0.3; 2.7)
Arterial lactate [mmol/L]	Control	1.4 (1.1; 1.7)	1.9 (1.4; 1.9)
	ELS	1.7 (1.4; 2.3)	2.0 (1.6; 2.6)
Arterial glucose [mg/dL]	Control	85 (79; 90)	86 (79; 91)
	ELS	93 (89; 104)	102 (92; 109) **p = 0.066**
Arterial Na^+^ [mmol/L]	Control	144 (143; 144)	143 (142; 144)
	ELS	142 (142; 143)	144 (143; 145)
Arterial K^+^ [mmol/L]	Control	3.6 (3.5,3.7)	3.8 (3.6; 3.9)
	ELS	3.6 (3.5; 3.8)	3.5 (3.4; 3.6)

**TABLE 2:** Requirements for fluid resuscitation and circulatory support in the control and Early Life Stress (ELS) groups (n = 6 each). “Arrhythmia” refers to the number of animals presenting with cardiac arrhythmia, i.e., ventricular extrasystoles and/or atrio-ventricular block II°, *Akrinor* ^**®**^ is a combined solution of theodrenaline/cafedrine 10/200 mg/mL; all data are median (interquartile range); the bold number refers to the *p*-value for intergroup comparison.

Colloids [mL/kg BW]	Control	5.8 (5.0; 7.2)
	ELS	7.4 (5.7; 10.6)
Akrinor^®^ [µL/kg BW]	Control	6.4 (5.2; 12.9)
	ELS	17.3 (16.3; 24.9) **p = 0.071**
Atropine [µg/kg BW]	Control	3.8 (2.6; 6.8)
	ELS	6.3 (3.5; 7.3)
Number of animals with cardiac arrhythmia	Control	1 (♂)
	ELS	3 (♀:1; ♂: 2)

**TABLE 3:** Blood cell count in the control (n = 5) and Early Life Stress (ELS) groups (n = 6). Data are median (interquartile range).

Red blood cells [Tera/L]	Control	5.1 (5.1; 5.4)
	ELS	4.8 (4.4; 5.3)
Total white blood cells [Giga/L]	Control	13.7 (10.1; 13.8)
	ELS	14.1 (12.6; 15.9)
Platelets [Giga/L]	Control	193 (192; 233)
	ELS	253 (234; 267)

**FIGURE 1:**
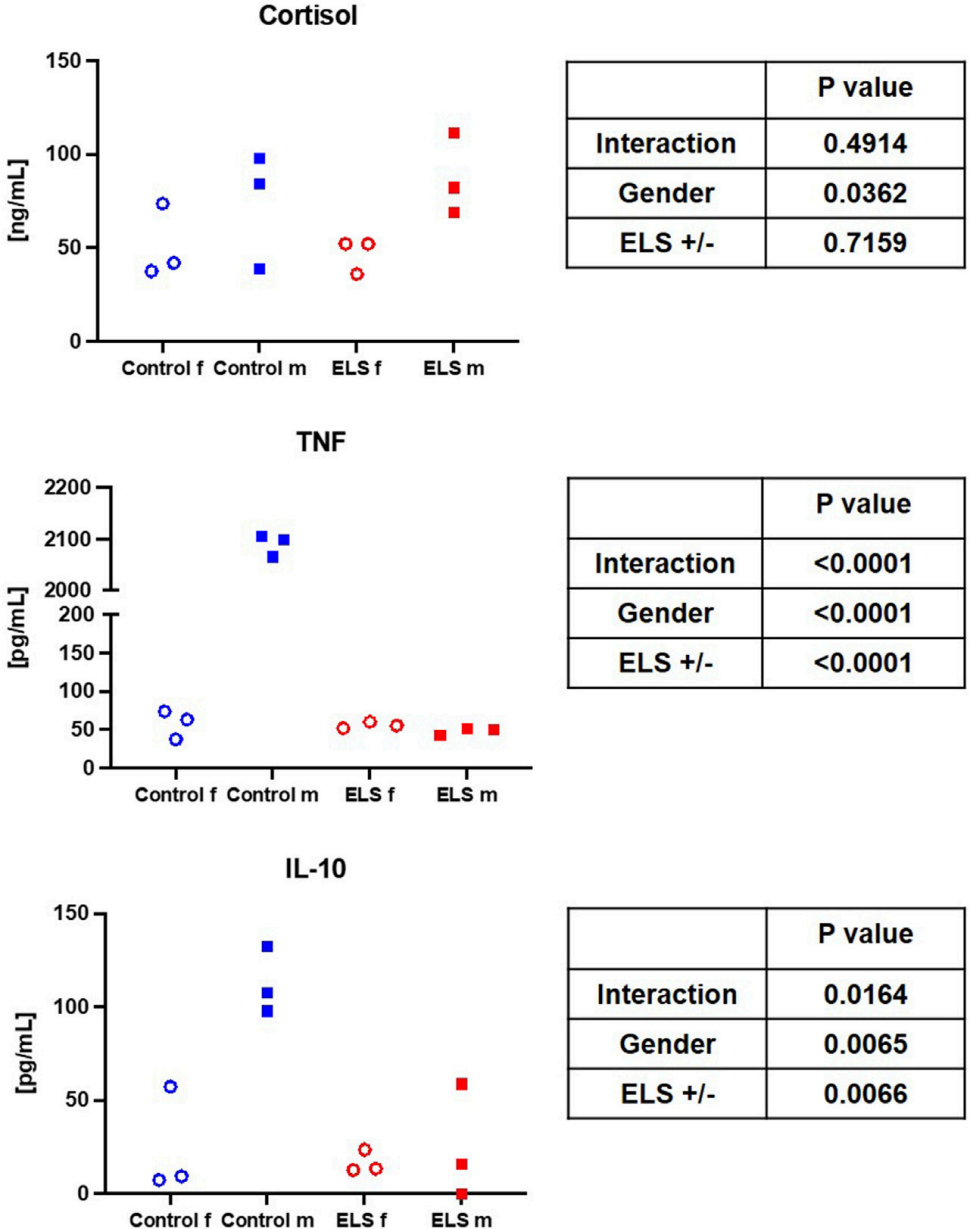
Serum cortisol, tumor necrosis factor (TNF, left panel) and interleukin-10 (IL-10, right panel) levels in the in the control (blue symbols) and Early Life Stress (ELS; red symbols) groups separated for male (“m”, solid squares) and female (“f”, open circles) swine. Note that male control animals showed markedly higher cytokine concentrations than females, while there was no sex-related difference in the ELS swine.

**FIGURE 2:**
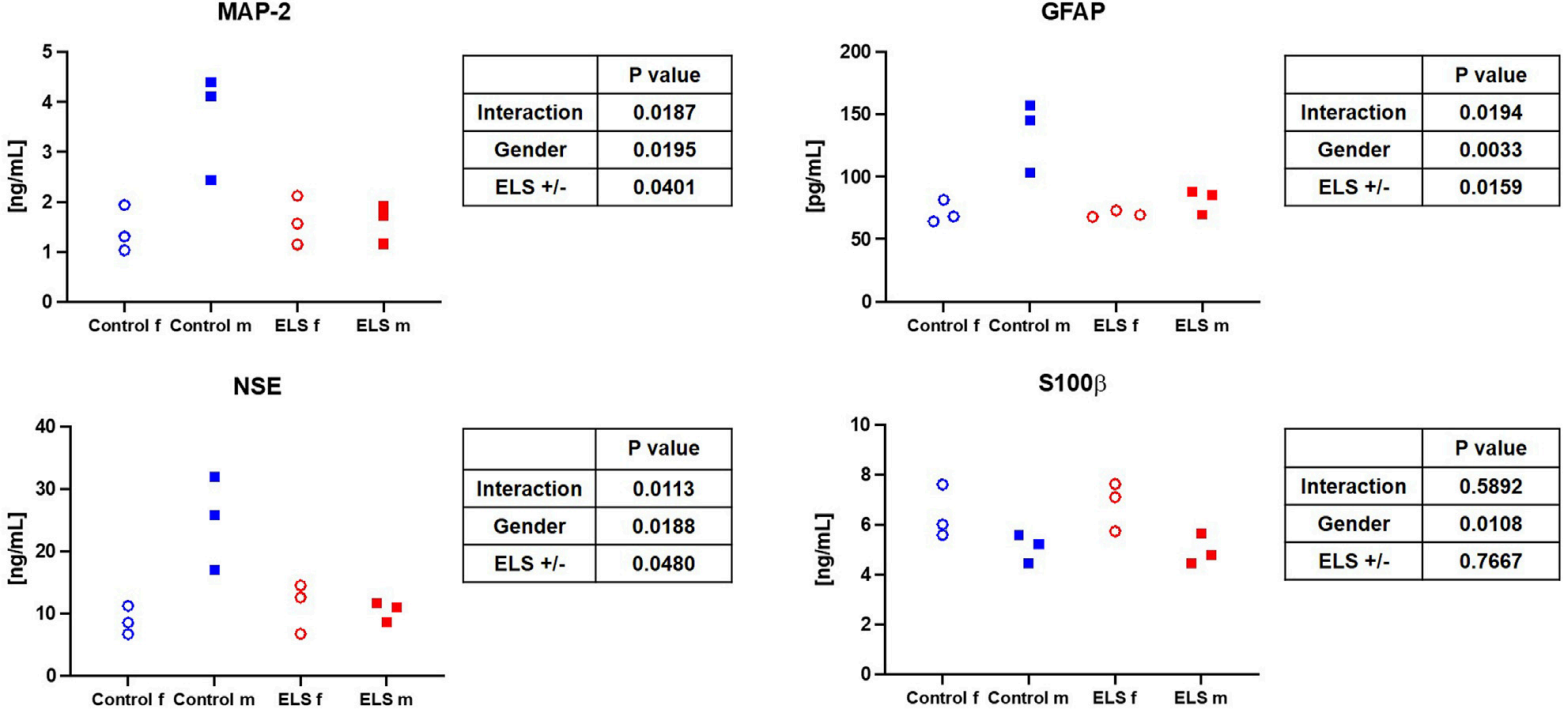
Serum microtubule-associated protein 2 (MAP-2) (upper left panel), glial fibrillary acidic protein (GFAP) (upper right panel), neuron-specific enolase (NSE) (lower left panel) and protein S100β (lower right panel) in the control and Early Life Stress (ELS) groups for the control (blue symbols) and Early Life Stress (ELS; red symbols) groups separated for male (“m”, solid squares) and female (“f”, open circles) swine. Note that male control animals showed markedly higher “serum brain injury marker” concentrations than females, while there was no sex-related difference in the ELS swine.

Table 4 and Figures 1, 2 summarize the results of the analysis of the serum stress, inflammation, and brain injury markers. There was no intergroup difference in catecholamine, cortisol, ACTH, renin, aldosterone, IL-6, and 8-isoprostane concentrations. However, ELS animals presented with both lower TNFα and IL-10 levels, but this difference was entirely due to the fact that male control swine had higher TNFα levels compared to the female animals, a sex-specific difference that was absent in the ELS animals (Figure 1). Overall, there was no difference in the “brain injury markers” MAP-2, GFAP, NSE, S100β either, but MAP-2, GFAP, and NSE levels were markedly higher in male controls than in male ELS pigs (Figure 2) and S100β levels were generally lower in male animals than female animals.

**TABLE 4:** Plasma concentrations of catecholamines (adrenaline, noradrenaline), cortisol, adrenocorticotropic hormone (ACTH), renin, aldosterone, cytokine (tumor necrosis factor (TNF), interleukin-6 and -10 (IL-6 and -10)), 8-isoprostane and “serum brain injury markers” (microtubule-associated protein 2 (MAP-2), glial fibrillary acidic protein (GFAP), neuron-specific enolase (NSE), protein S100β (S100β)) and testosterone in the control and Early Life Stress (ELS) groups (n = 6 each). All data are median (interquartile range) except for testosterone (median (minimum and maximum)).

Adrenaline [pg/mL]	Control	58 (22; 90)
	ELS	106 (54; 251)
Noradrenaline [pg/mL]	Control	123 (79; 161)
	ELS	196 (102; 329)
Cortisol [ng/mL]	Control	58 (40; 82)
	ELS	61 (52; 79)
ACTH [ng/mL]	Control	3.4 (1.7; 3.8)
	ELS	3.7 (2.8; 5.6)
Renin [pg/mL]	Control	9 (8; 11)
	ELS	9 (7; 15)
Aldosterone [pg/mL]	Control	405 (375; 420)
	ELS	379 (342; 433)
TNF [pg/mL]	Control	1,070 (66; 2091)
	ELS	52 (50; 55) **p = 0.048**
IL-6 [pg/mL]	Control	10 (7; 19)
	ELS	7 (3; 14)
IL-10 [pg/mL]	Control	78 (21; 105)
	ELS	15 (13; 22) **p = 0.063**
8-isoprostane [pg/mL]	Control	47 (44; 52)
	ELS	40 (37; 43)
Troponine [ng/mL]	Control	0.021 (0.015; 0.031)
	ELS	0.021 (0.016; 0.040)
S100β [ng/mL]	Control	5.6 (5.3; 5.9)
	ELS	5.7 (5.0; 6.8)
MAP 2 [ng/mL]	Control	2.2 (1.5; 3.7)
	ELS	1.6 (1.3; 1.9)
GFAP [pg/mL]	Control	92 (72; 135)
	ELS	71 (69; 82)
NSE [ng/mL]	Control	14.2 (9.2; 23.6)
	ELS	11.3 (9.2; 12.4)
Testosterone [ng/mL]	Control ♀	0.8 (0.7; 0.9)
	ELS ♀	0.62 (0.6; 0.7)
	Control ♂	1.1 (1.0; 1.7)
	ELS ♂	1.3 (1.1; 1.4)

**FIGURE 3:**
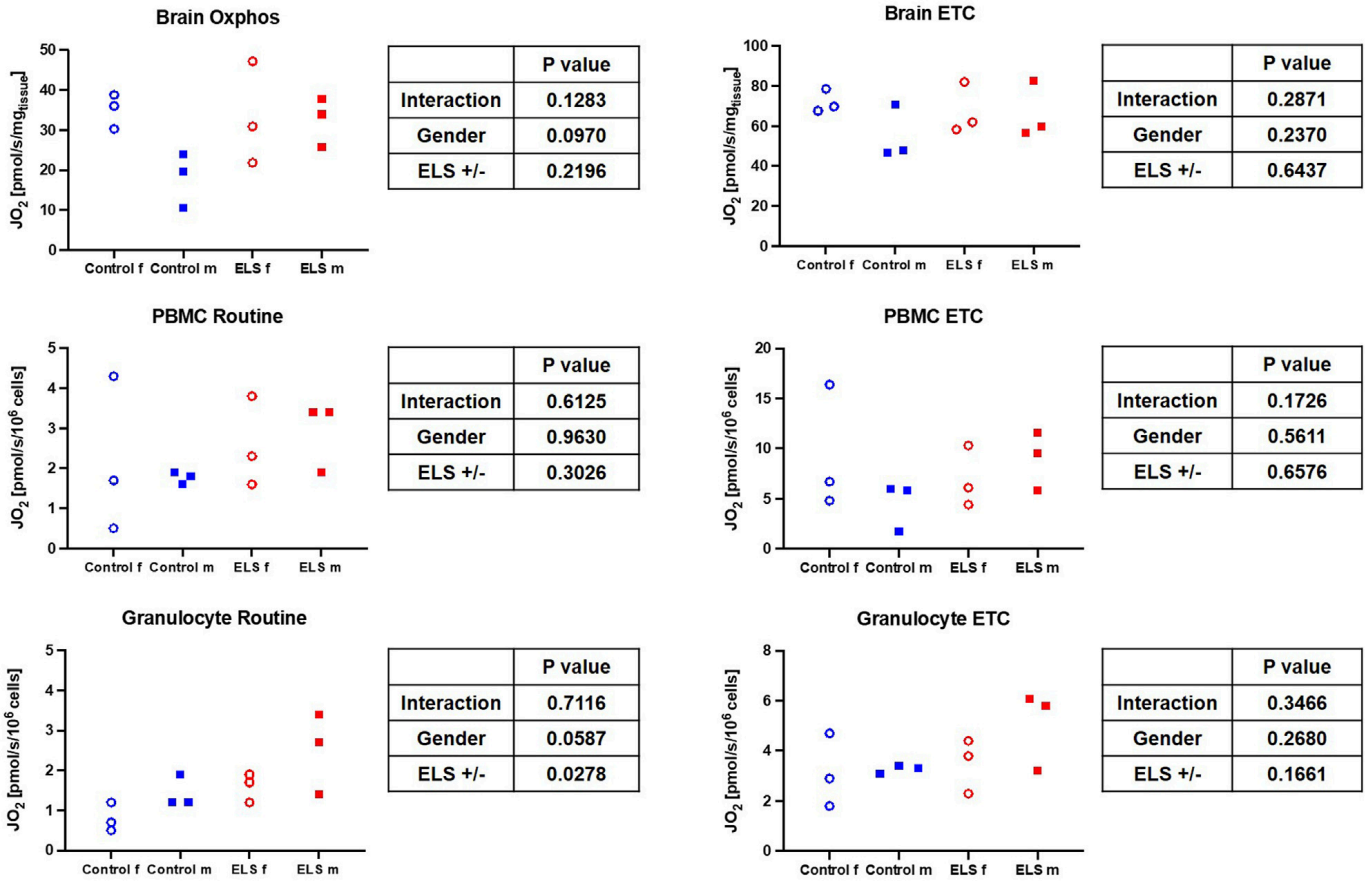
Mitochondrial respiration (left column: oxidative phosphorylation (OxPhos)/physiological coupling state (Routine); right column: maximum electron transport capacity of the respiratory chain in the uncoupled state) in immediate *post mortem* brain specimen (J*O*_*2*_ [pmol/s/mgtissue]) (upper panel) as well as in isolated peripheral blood mononuclear cells (PBMC, middle panel) (J*O*_*2*_ [pmol/s/10^6^ cells]) and granulocytes (lower panel) J*O*_*2*_ [pmol/s/10^6^ cells(]) for the control (blue symbols) and Early Life Stress (ELS; red symbols) groups in male (solid squares) and female (open circles) swine. Note that as expected, ETC., in PBMC was markedly higher than in granulocytes.

Table 5 and Figure 3 summarize the results of the high-resolution-respirometry measurements in the immediate *post mortem* brain tissue specimen as well as in the isolated PBMC and granulocytes. Overall, the measured parameters of mitochondrial respiratory activity did not differ between control and ELS animals (Table 5; Figure 3), except for significantly lower granulocyte routine respiration in the control pigs. Plotting the brain tissue, ETC., as a function of the, ETC., of PBMC showed no significant relation (*p* = 0.173), irrespective of the presence/absence of ELS. A similar result was found when brain tissue, ETC., was plotted as a function of the PBMC bioenergetic health index (BHI) (*p* = 0.555).

**TABLE 5:** Mitochondrial respiration (J*O*_2_) in isolated peripheral blood mononuclear cell (PBMC) and granulocytes (in [pmol/s/10^6^cells]) as well as in immediate *post mortem* brain specimen (in [pmol/s/mg_tissue_]) in the control and Early Life Stress (ELS) groups (n = 6 each). OxPhos: oxidative phosphorylation, Routine: physiological coupling state, ETC.,: electron transport capacity of the respiratory chain in the uncoupled state; all data are median (interquartile range).

		OxPhos	Routine	ETC
Brain [pmol/s/mg_tissue_]	Control	27 (21; 35)		69 (53; 71)
	ELS	32 (27; 37)		61 (59; 77)
PBMC [pmol/s/10^6^cells]	Control		2 (2; 2)	6 (5; 7)
	ELS		3 (2; 3)	8 (6; 10)
Granulocytes [pmol/s/10^6^cells]	Control		1 (1; 1)	3 (3; 3)
	ELS		2 (2; 3) **p = 0.039**	4 (3; 5)

**FIGURE 4:**
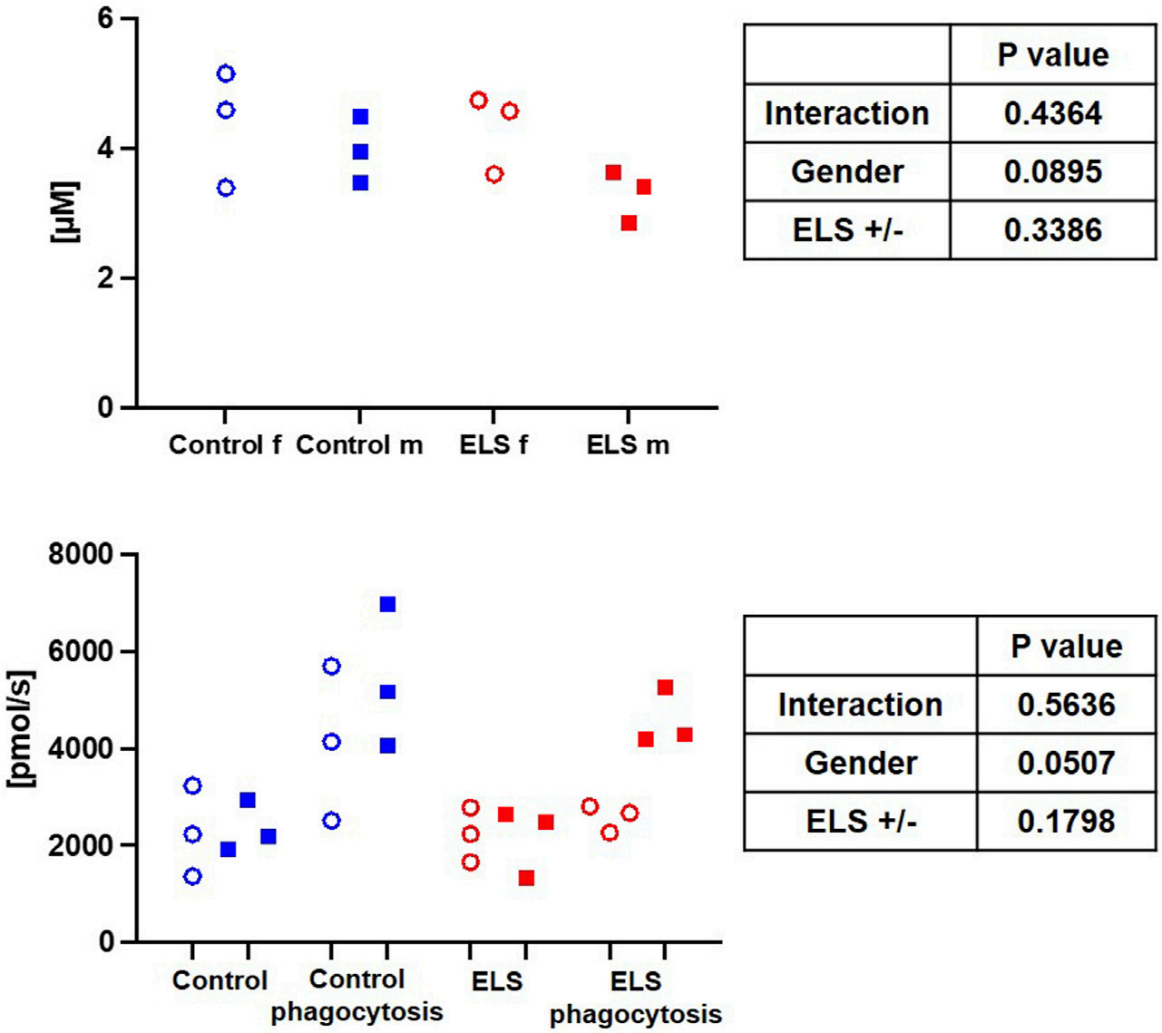
Whole blood superoxide anion (O_2_^•^¯) concentration in the control (blue symbols) and Early Life Stress (ELS; red symbols) groups separated for male (“m”, solid squares) and female (“f”, open circles) swine (upper panel). Granulocyte superoxide anion (O_2_^•^¯) production at baseline and after stimulation of phagocytosis with *E. coli* in control (blue symbols) and Early Life Stress (ELS; red symbols) animals separated for male (solid symboles) and female (open symboles) swine (lower panel). Each symbol refers to samples from an individual animal. Note that O_2_^•^¯ release increased upon stimulation in all samples from male swine irrespective of the presence/absence of ELS, while this response was abolished in female animals with ELS.

Table 6 and Figure 4 show the results of the electron spin resonance measurements for the quantification of the O_2_^•^¯ formation. Both whole blood O_2_^•^¯ concentrations immediately after sampling (Figure 4) and O_2_^•^¯ production at baseline and after stimulation with *E. coli* particles in isolated PBMC were comparable, no matter the gender and/or group assignment of the animals (Table 6). In contrast, granulocyte O_2_^•^¯ production after stimulation with *E. coli* particles tended to be lower in the ELS group (Table 6), and this effect was due to the response pattern in female swine: while isolated granulocytes of both females and males of the control group showed increased O_2_^•^¯ production upon stimulation with *E. coli* particles, this response was abolished in the female ELS animals (Figure 4).

**TABLE 6:** Peripheral blood mononuclear cell (PBMC) and granulocyte superoxide anion (O_2_^•^¯) production at baseline and after stimulation of phagocytosis with *E.coli* in the control (n = 5) and Early Life Stress (ELS) groups (n = 6). Data are median (interquartile range); the bold number refers to the *p*-value for intergroup comparison.

PBMC [nmol/sec/10^6^ cells]	Control	0.07 (0.05; 0.09)
	ELS	0.06 (0.05; 0.07)
Granulocytes [nmol/sec/10^6^ cells]	Control	2.2 (2.0; 2.8)
	ELS	2.4 (1.8; 2.6)
PBMC-Phagocytosis [nmol/sec/10^6^ cells]	Control	0.10 (0.03; 0.13)
	ELS	0.15 (0.14; 0.17)
Granulocytes-Phagocytosis [nmol/sec/10^6^ cells]	Control	4.7 (4.1; 5.6)
	ELS	3.5 (2.7; 4.3) **p = 0.086**

## 4 Discussion

The aim of this study was to investigate the effects of early weaning as a model of Early Life Stress (ELS) on physiological parameters and mitochondrial respiratory capacity of circulating blood cells as well as the brain in 6-month old anesthetized pigs. The main findings were that *i*) at sexual maturity, ELS may affect both the immune response to general anesthesia as well as O_2_^•^¯ radical production in a gender-specific manner, and that *ii*) the mitochondrial respiratory capacity of the brain is not reflected by peripheral blood immune cells.

This porcine model of early weaning of piglets at 21 days after birth induces ELS to the mildest possible degree. This weaning time point had been chosen to comply with the Federal German regulations on farm animal husbandry, and, according to literature reports, it is the latest possible early weaning to induce a stress phenotype in the intestine (Smith et al., 2010). Interestingly, ELS and control swine did not show any difference with respect to growth, body weight, and behavior. This very mild stress was associated with a higher occurrence of cardiovascular complications in response to general anesthesia in sexually mature pigs: 3 out of 6 animals in the ELS group displayed ventricular extrasystole and/or atrio-ventricular block II°, *versus* only 1 out of 6 animals within the control group (see Table 2). Additionally, ELS pigs presented with a lower MAP despite higher circulatory support (see Tables 1, 2). These findings suggest that this very mild ELS model may mirror patients suffering from a higher cardiovascular risk as a consequence of ELS (Felitti et al., 1998; Suglia et al., 2020; McCook et al., 2021). Furthermore, pigs in the ELS group exhibited higher glycemia levels compared to the control group (see Table 1). These results are in accordance with literature reports: study participants (sex-mixed, 65% female) with a history of childhood maltreatment (CM), in other words ELS, presented with significantly higher 1-h glucose levels in the oral glucose tolerance test as well as slightly elevated glucose levels at baseline (Li et al., 2017). Furthermore, that study reported a direct linear correlation of elevated TNFα levels with CM scores. Interestingly, in contrast to these findings, in our study we determined significantly lower TNFα levels in the ELS group compared to control animals (see Table 4). This difference, is mostly related to a pronounced sex-specific difference in TNFα levels in the control group: male animals had several-fold higher TNFα levels than females (see Figure 1). Female ELS animals had similar TNFα levels than female controls, and there was no difference in TNFα levels between male and female animals in the stress group. We observed a similar pattern for IL-10. Literature reports on baseline levels of cytokines (with general anesthesia, but no additional stimulation), sex specific differences and/or the effects of stress on cytokine levels are very scarce if available at all.

Bernardi *et al.* reported higher TNFα levels in male compared to female healthy adults, which is in line with our results (Bernardi et al., 2020). In contrast to our study, however, these authors also found elevated IL-6 levels in males, but no differences in IL-10 (Bernardi et al., 2020). It is noteworthy in this context that the authors overall reported much lower mean cytokine levels than in our study, suggesting that general anesthesia and the surgical instrumentation *per se* may have led to an induction of cytokine expression, which has also been previously reported in the literature (Bauer et al., 1998). In our study, this anesthesia-related induction of cytokines was most pronounced in male control animals. Lahat *et al.* investigated TNFα levels in male patients undergoing coronary artery bypass graft surgery or cholecystectomy, which were elevated in all patients in contrast to healthy controls, and even further elevated with general anesthesia (Lahat et al., 1992). Anesthesia did not stimulate IL-6 levels in their study, and IL-10 was not investigated. The TNFα levels reported by Lahat *et al.* are drastically higher than the levels reported by Bernardi *et al.*, with levels from our study falling in between the two literature reports. Unfortunately, the study by Lahat *et al.* focused on male patients, so sex-specific differences were not investigated. In accordance to our findings, levels of TNFα were higher in male pigs than in female swine after resuscitation from cardiac arrest, and this effect was not related to steroid hormones (Niemann et al., 2008). In light of our results taken together with literature reports, it is tempting to speculate that males might react more severely to a first hit, such as anesthesia and surgery, than females.

Reports on the effect of ELS alone on systemic cytokine levels are inconsistent, as reviewed in a recent meta-analysis (Dutcher et al., 2020). Interestingly, in our study, ELS prohibited the anesthesia-induced elevation of TNFα and IL-10 in male animals. This partially confirms results from a rodent model of ELS, where a transient significant decrease in IL-10 levels was reported in stressed male animals compared to the control group (Grassi-Oliveira et al., 2016). In females, however, IL-10 levels did not significantly differ between control and stressed animals (Grassi-Oliveira et al., 2016). The transient nature of the impact of ELS on cytokine levels might help to explain why most studies report no effect of ELS on TNFα, IL-10 or IL-6 expression (Dutcher et al., 2020). ELS in combination with a forced swimming test (considered a “*second stress*” or second hit) was associated with reduced IL-10 levels and increased TNFα in male rats (Réus et al., 2013), which at least partially agrees with our findings (if the induction of general anesthesia and catheterization of the left iliacal artery in our study is considered a “*second stress*”). The lack of an effect of stress on IL-6 on the other hand is in accordance with a literature report from a model with ELS and ovalbumin challenge in male rats (Kruschinski et al., 2008).

In contrast to our findings, in healthy male volunteers with CM the increase of cytokine levels after the Trier Social Stress Test (TSST) was more pronounced than in the no-CM group (Shalev et al., 2020). The cytokine response is largely regulated by cortisol, which also displayed a significantly higher increase in the CM group in male healthy volunteers (Shalev et al., 2020). In contrast, in our study there was no difference in cortisol levels between animals with and without ELS (see Figure 1), which is partially in line with literature reports from Young *et al.* and Zhang *et al.*: blunted cortisol response to acute stress in women (Young et al., 2021) and lower baseline cortisol levels and blunted cortisol response to acute stress (i.e., a TSST) in patients (male and female) with severe CM (Zhang et al., 2019). Interestingly, the effect of stress on cortisol seems to depend on the stress load: in patients with moderate CM, the cortisol response was actually more pronounced than in the control group (Zhang et al., 2019). It is worthy of note that general anesthesia also induces cortisol release (Ishihara et al., 1979), which might be masking the effects of ELS on the cortisol response in our study.

In our study, the only difference in mitochondrial activity between control and ELS animals was in the routine respiration activity of granulocytes, which was significantly lower in the control than in the ELS pigs (see Figure 3). There was no intergroup-difference in mitochondrial respiratory capacity in PBMCs, which is in part in contrast to the literature: women with CM had significantly higher PBMC routine respiration 1 week and 3 months *postpartum*, however there was no difference between mothers with and without CM 1 year *postpartum* (Boeck et al., 2016; Gumpp et al., 2020; Gumpp et al., 2022). Furthermore, there is evidence in the literature that ELS is associated with elevated blood mitochondrial DNA content in adults (sex-mixed, 61% female) (Ridout et al., 2020). The authors of a recent review suggest that this is a compensatory mechanism to counteract ELS-induced mitochondrial dysfunction and/or increased energy demand (Zitkovsky et al., 2021). In context with the findings of the very limited effects of ELS on mitochondrial function, they further suggest that “*alterations in mtDNAcn (might) occur independently to changes in mitochondrial function*” (Zitkovsky et al., 2021), which is in line with our results on mitochondrial respiratory capacity in PBMCs. Unfortunately, we could not determine mtDNA content in our study. Glucocorticoid signaling has been suggested as a molecular mechanism for stress-induced alterations in the mitochondria (Ridout et al., 2020); however, neither we nor Böck *et al.* found any association between cortisol levels and PBMC oxygen consumption (see Figures 1, 3; Boeck et al., 2018).

Immune cell bioenergetics can also impact ROS production (Chance et al., 1979). We did not find any intergroup difference in whole blood levels of ROS; however, in the control group, there was a trend towards higher ROS production in granulocytes stimulated with *E. coli*. This effect was even more pronounced in male ELS animals, which also had the highest granulocyte routine respiration. Interestingly, in granulocytes from female stressed animals, the *E. coli*-stimulated ROS release was abolished, suggesting a functional impairment or lack of responsiveness of these cells.

Literature reports on sex-specific differences on (cerebral) mitochondrial function and/or how it is affected by ELS are very limited. Silaidos *et al.* reported sex-specific differences in the mitochondrial respiration of PBMCs: in contrast to our results, where we did not see sex-specific difference in the mitochondrial respiration of PBMCs, cells from male healthy volunteers displayed significantly lower routine respiration compared to cells derived from female study participants (Silaidos et al., 2018). Furthermore, using magnetic resonance imaging, the authors detected a higher cerebral energy consumption in female brains (Silaidos et al., 2018). We also determined a trend towards a lower respiratory capacity under OxPhos conditions in male animals compared to female animals in our control group (see Figure 3); thus, it is tempting to speculate that the higher cerebral energy consumption reported by Silaidos *et al.* might also be related to higher cerebral mitochondrial respiration in women. Interestingly, the lower mitochondrial respiration in male control animals was associated with higher levels of systemic “brain injury” markers, i.e., MAP-2, GFAP and NSE (see Figure 2). Our data partially confirm the results from a rodent study of Gonzales-Pardo *et al.*, who also observed higher mitochondrial activity in samples derived from female compared to male brains (González-Pardo et al., 2020). However, their reported stress-induced decreased mitochondrial function is in contrast to our results. Another rodent model of ELS also revealed mitochondrial dysfunction, evidenced by lower ATP content, and neuroinflammation in the hippocampus of male mice (Amini-Khoei et al., 2017). The degree of ELS in these rodent models might be more severe than in our early weaning large animal model, where we found very limited effects of ELS on the mitochondrial respiratory capacity in the brain; however, male ELS animals had slightly higher mitochondrial capacity than male control animals, whereas in female animals it remained unaffected (see Figure 3). This is in contrast to the findings of Amini-Khoei *et al.*, but well agrees with the suggested increased energy demand under stress conditions discussed above. Due to the very limited availability of brain samples and non-invasive methods to study the brain, it is very difficult to assess effects of CM on cerebral mitochondrial function in humans. Thus, most clinical studies rely on the investigation of PBMCs, which are easily accessible. This begs the question whether mitochondrial respiratory activity of PBMCs can serve as a surrogate for mitochondrial function in less accessible tissues, e.g., the brain. Two studies in non-human primates (female African green monkeys) determined a direct linear relation between the so-called monocyte “bioenergetic health index (BHI)” and the maximum respiratory activity in cerebral frontal cortex mitochondria or maximum monocyte respiration with skeletal muscle mitochondrial respiration (Tyrrell et al., 2017; Tyrrell et al., 2016). We could not confirm these results: in our study, there was no significant relation between PBMC maximum respiration or BHI with cerebral frontal cortex mitochondrial activity. This is in line with the findings from Silaidos *et al.*, who did not observe either any significant correlation between parameters of cerebral energy consumption and PBMC bioenergetics (Silaidos et al., 2018).

### 4.2 Limitations

Our study is limited by the fact that for this pilot experiment, we were only able to obtain a permission from the Animal Care Committee of the Universität Ulm and the Federal Authorities for Animal Research (Regierungspräsidium Tübingen) for six control and six ELS animals. Therefore, a power calculation was unavailable. More significant differences may have been missed due to the small number of pigs in each group.

### 4.3 Translational relevance of the porcine early weaning model

Rodent models of ELS, predominantly mouse models with maternal separation, i.e., separation of the mother from her pups for several hours per day (normally 3 h) on repeated days (normally postnatal day 1 though postnatal day 14–21) are most commonly used in ELS research. However, many contradictory and inconsistent reports on the results of rodent models and human studies can be found in the literature (Murthy and Gould, 2018). We and others are speculating, that this might partially be due to differences in rearing behavior of rodents and humans. The time a child spends with their parents is normally much longer and much more sociable in humans than in mice, even normalized for the according life span. In many relevant aspects for early life stress models; i.e., longer gestation period, longer rearing of young animals and social behavior; large mammals, such as pigs, are much more comparable to humans than rodents. Furthermore, rodent development is characterized by a stress hyporesponsive period, which is not the case in humans (Parker and Maestripieri, 2011). The anatomy and physiology of pigs is very similar to humans, which makes pathophysiological investigations in pigs much more translationally relevant compared to rodents. Furthermore, the process of weaning itself in rodents is much shorter than in pigs and humans. This also implies, that the vulnerable phase, during which the young animal/child does not only need the parents to provide them with food (which can be taken over by other means after weaning) but also for emotional support and caretaking (for which the primary caregiver cannot be replaced) is much shorter in rodents than in large mammals and humans. Due to the longer and more socially shaped rearing period in pigs, we postulate a better translational comparability of the pathophysiology of early life stress with the human situation, even though the data on stress vulnerability *versus* resilience for large animals and pigs are very scarce. However, it is known from human studies, that the time-point weaning of babies and/or their separation from the primary caregiver and even small changes in this time-point (<1month) can have long-term effects on their (mental) health (Hayatbakhsh et al., 2012; Loret de Mola et al., 2016). In that regard, the porcine early weaning model used in this study represents a translationally relevant model of ELS because differences in the pig model depending on the length of weaning/separation are clearly more comparable to humans than similar investigations in the mouse model. An early separation from the primary caregiver, as used in the model presented here, is considered emotional neglect, which is, along with physical neglect and emotional, sexual and physical abuse, a recognized type of Childhood Maltreatment in humans.

## 5 Conclusion

In our study, ELS was associated with a higher prevalence of cardiac arrhythmia, altered inflammatory response to general anesthesia and slight mitochondrial aberrations. Our data suggest sex-specific differences in the immunological and mitochondrial response to ELS. Male stressed animals were characterized by an ameliorated upregulation of cytokines in response to anesthesia, the strongest induction of ROS production by granulocytes stimulated with *E. coli*, and a trend towards upregulated mitochondrial respiration. In contrast, in female animals, these parameters remained largely unaffected, except for the abolished induction of *E. coli*-stimulated granulocyte ROS release of stressed female animals. These results might suggest that ELS is a form of stress pre-conditioning, which is more efficient in females than males. Taken together with assumptions that female mitochondria are more resilient to stress than those of males (Demarest and McCarthy 2015), it is tempting to speculate that the biological response to stress in general is more pronounced in males than females.

## Data Availability

The raw data supporting the conclusions of this article will be made available by the authors, without undue reservation.
